# Role and relevance of genetic testing in patients with kidney stones: a review from EAU Section of Endourology

**DOI:** 10.1097/MOU.0000000000001420

**Published:** 2026-06-17

**Authors:** Carsten A. Wagner, John A. Sayer, Michael Straub

**Affiliations:** a Institute of Physiology and Zurich Kidney Center, University of Zurich, Switzerland; b ESEUT European Association of Urology Endourology Section; c Biosciences Institute, Newcastle University, Central Parkway; d Renal Services, The Newcastle upon Tyne NHS Foundation Trust; e National Institute for Health Research Newcastle Biomedical Research Centre, Newcastle upon Tyne, UK; f TUM University Hospital Rechts der Isar, Department of Urology, Endourology and Stone Center, Munich, Germany

**Keywords:** chronic kidney disease, cystinuria, genome-wide association studies, high risk, kidney stone disease, monogenic disease, recurrent kidney stones, SLC34 transporters

## Abstract

**Purpose of review:**

Kidney stones have a high heritability. More than 40 genes have been identified causing monogenic forms of kidney stone disease (KSD). Kidney stone formers with genetic variants implicated in monogenic forms of KSD often suffer from early onset, high recurrence rates, and chronic kidney disease. Some patients may also exhibit extrarenal disease requiring attention.

**Recent findings:**

Recent analysis of KSD patients identified a likely monogenic cause in pediatric populations in 17–30% of participants while in adult unselected populations 2.7–8% had a positive finding. More patients carry single genetic variants in monogenic forms that are classically considered as autosomal recessive but may cause an intermediate genetic risk for the development of KSD possibly in interaction with environmental or lifestyle factors. Genome-wide association studies have identified additional risk loci associating with KSD. Their clinical relevance are currently investigated. Patients with recurrent kidney stone episodes may be at elevated risk of progressive chronic kidney disease.

**Summary:**

Monogenic causes of KSD are prevalent in patients less than 25 years of age and in some patients with high-risk metabolic profiles. These patients should undergo genetic testing to enable a precise molecular genetic diagnosis and personalized therapy as well as family counseling and screening.

## INTRODUCTION

Kidney stone disease (KSD) affects approximately 10–12% of the worldwide population [1]. While historically more prevalent in male individuals, recent epidemiological shifts show a narrowing gender gap, driven by a steeper rise in adolescent female cases [2]. Globally, KSD prevalence varies regionally, influenced by dietary habits, climate, and socioeconomic factors, though mechanistic drivers remain incompletely understood.

The pathogenesis of KSD is multifactorial with important contributions from metabolic, environmental, and genetic factors. Key contributors include urinary supersaturation of calcium oxalate, uric acid, or struvite; acidic or alkaline urine pH; and anatomic abnormalities [1,2]. Obesity, type 2 diabetes mellitus, and chronic antibiotic use further modulate risk, with sex-specific patterns: men exhibit higher urinary calcium/oxalate excretion and uric acid supersaturation, whereas women face greater quality-of-life impairments and postprocedural sepsis risks [2]. Hormonal influences may partly explain these disparities.

Heritability studies reveal a strong genetic component to KSD, with twin analyses estimating 46% heritability in women and 57% in men [3,4]. Classic twin studies demonstrate striking genetic control over urinary biomarkers: calcium (94%), oxalate (94%), citrate (95%), and brushite supersaturation (90%) exhibit near-complete heritability, while uric acid supersaturation (58%) and sodium excretion (64%) show stronger environmental modulation [5]. Somewhat lower heritability rates have been estimated for tubular transport processes in family-based population studies suggesting heritability to be in the range between 30 and 50% for sodium, potassium, phosphate, and calcium [6]. Nevertheless, these findings underscore genotype-driven tubular transport mechanisms, particularly in calcium/phosphate homeostasis and acid–base balance. Renal function itself has a heritability of 33% for estimated glomerular filtration rate (eGFR), exceeding genome-wide association study (GWAS) estimates, suggesting unaccounted epigenetic or gene–environment interactions [7].

This review synthesizes advances in genetic KSD research and discusses the use of genetic testing in patients with KSD to improve diagnosis, therapy, and family counseling. 

**Box 1:**
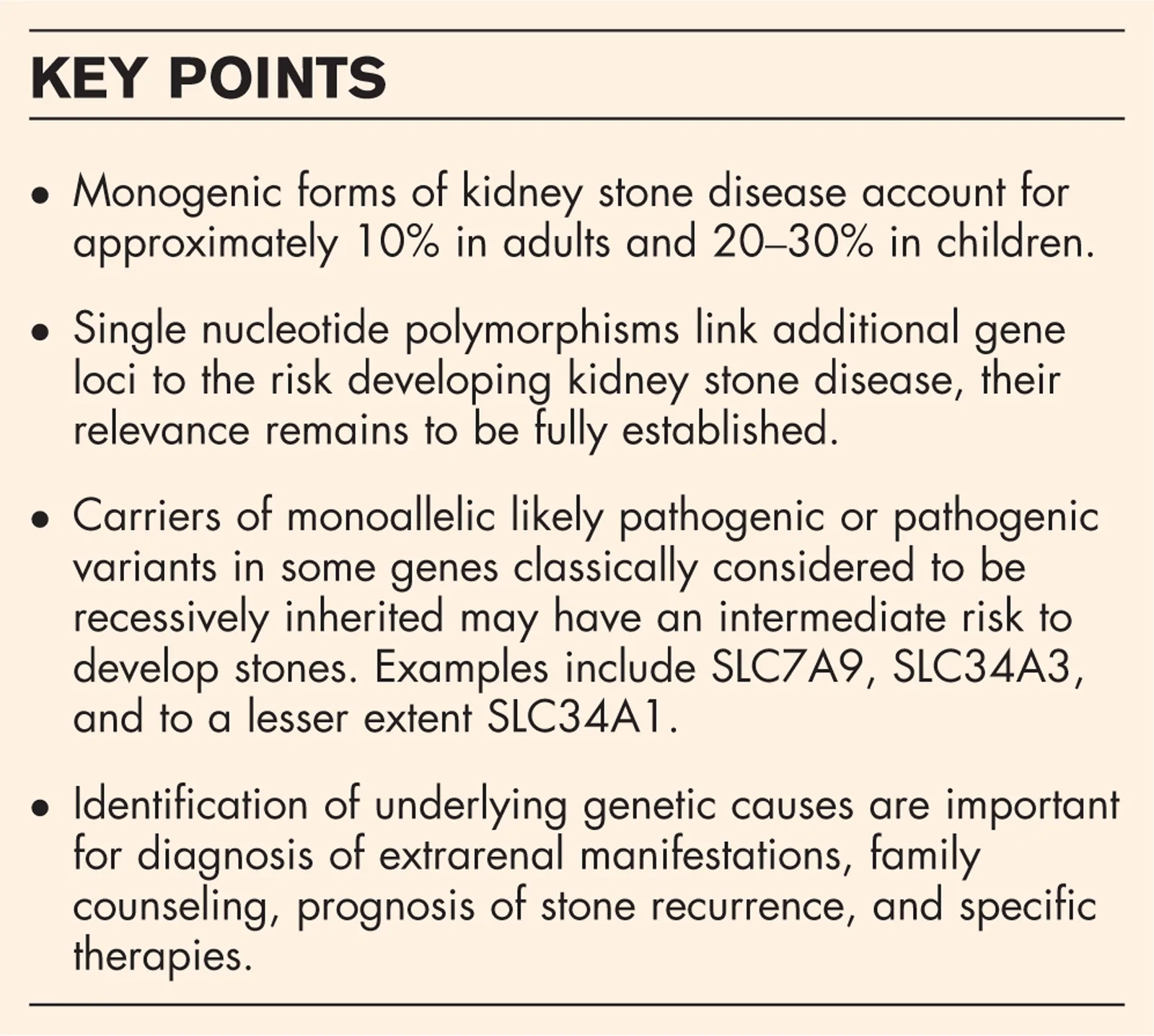
no caption available

## PREVALENCE OF MONOGENIC KIDNEY STONE DISEASE

Monogenic causes are estimated to account for approximately 10% of KSD cases. However, estimates vary among different populations, ranging from 17 to 30% in pediatric populations to 2.7–8% in unselected adult cohorts [8–12,13,14^▪▪^]. The positive diagnosis of monogenic kidney stone disease is uncertain due to new findings suggesting that genes previously thought to require biallelic variants (e.g. a recessive mode of inheritance) may also cause disease (with partial penetrance) when carrying only monoallelic variants (see below). Some of these monoallelic variants are common in the general population. For example, monoallelic coding variants in either *SLC34A1* or *SLC34A3* are present in 3–4% of the general population [15]. However, it is unclear whether all these variants can cause KSD, some may likely represent risk alleles, while others do not appear to associate with distinct biochemical abnormalities [14^▪▪^].

**FIGURE 1:**
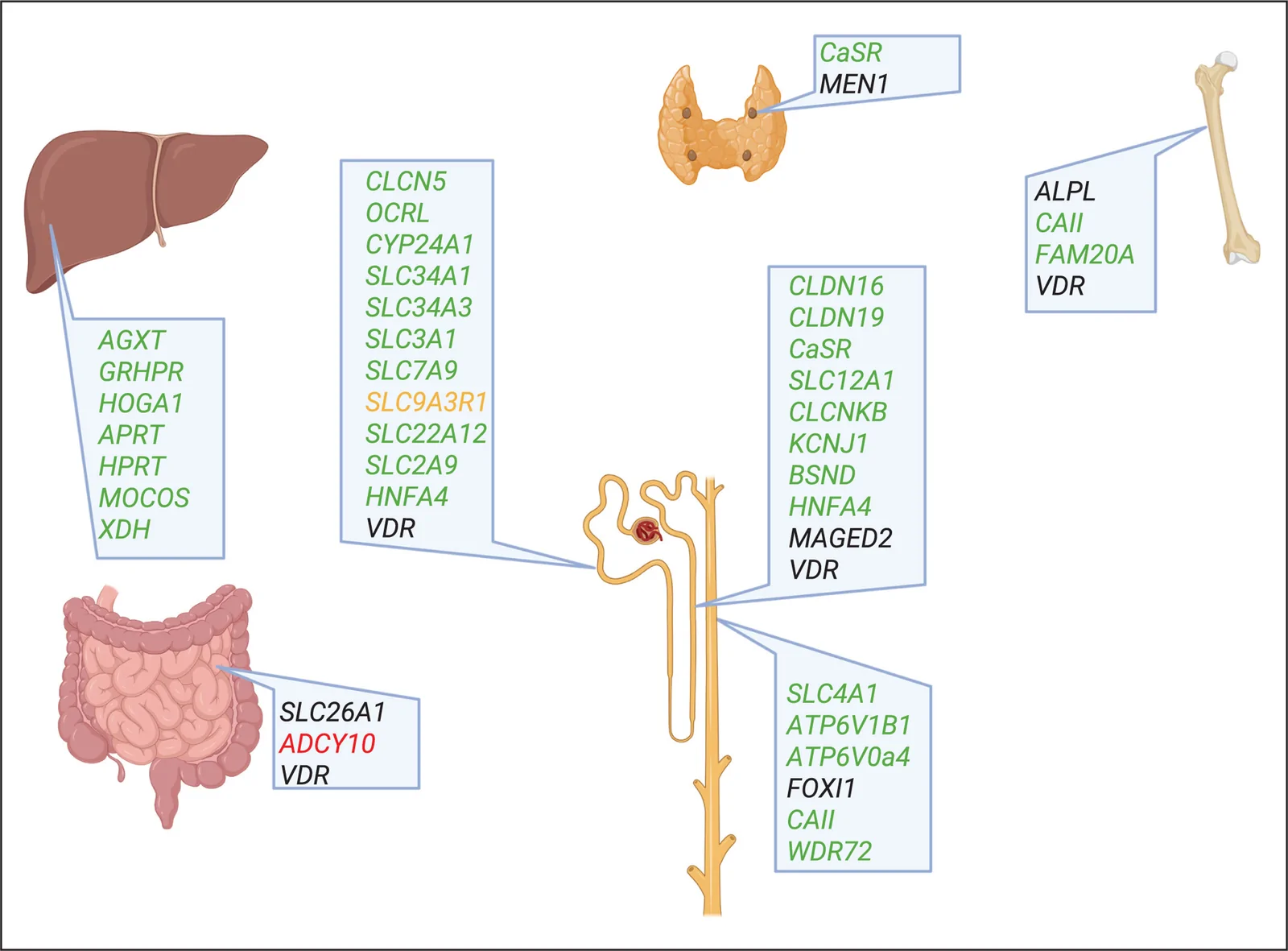
The major organs involved in the pathogenesis of kidney stone disease are the liver, small and large intestines, parathyroid glands, bones, and kidneys. Genes are listed which are involved in monogenic forms of kidney stone disease. Genes are grouped with the organs in which they are primarily expressed. The color code refers to the level of evidence as established for the genomic England app where green indicates recommendation by experts, amber unclear, and red no recommendation. Black indicates that no exert opinion is available yet.

Genes that cause causing monogenic KSD include genes critical for tubular electrolyte and mineral transport (i.e. *CLCN5*, *SLC34A1, SLC34A3*), paracellular transport (i.e. *CLDN16, CLDN19*), vitamin D metabolism (*CYP24A1*) or oxalate metabolism (*AGXT*) [16^▪^]. Many of these genes are expressed along the nephron, but important genes are also present in endocrine organs (i.e. parathyroid glands) or major metabolic organs (i.e. liver) (Fig. 1) and thereby directly or indirectly influence the concentration of stone forming solutes in urine.

Over 46 genes are now linked to Mendelian stone disorders (Table 1), with penetrance possibly influenced by dietary and comorbid factors [16^▪^]. Moreover, genome-wide association studies (GWAS) have confirmed the association of some of these genes with KSD in large population-based cohorts and identified numerous additional loci that might cause KSD [17]. A potential use of these genetic risk loci might be to incorporate them into the development of genetic risk scores predicting individual genetic risks for KSD and/or recurrence [16^▪^].

**Table 1:** Genes causing monogenic forms of kidney stone disease

Gene name	Protein name	Mode of inheritance/OMIM	Function	Reference
*ADCY10*	Soluble adenylate cyclase	AD 143870	Regulates intestinal calcium absorption	[18]
*AGXT*	Alanine-glyoxylate aminotransferase	AR 259900	Prevents oxalate accumulation; primary hyperoxaluria type 1	[19]
*ALPL*	Alkaline phosphatase	AD/AR 146300	Skeletal mineralization defect	[20]
*APRT*	Adenine phosphoribosyltransferase	AR 614723	Degrades 2,8-dihydroxyadenine	[21]
*ATP6V0A4*	V-ATPase a4 subunit	AR 602722	Urinary acidification, distal renal tubular acidosis (with sensorineural deafness)	[22]
*ATP6V1B1*	V-ATPase B1 subunit	AR 267300	Urinary acidification, distal renal tubular acidosis with sensorineural deafness	[23]
*BSND*	Barttin	AR 602552	Subunit of CLKNA and CLKNB chloride channels Bartter syndrome with sensorineural deafness	[24]
*CAII*	Carbonic anhydrase II	AR 259730	Osteopetrosis and intellectual disabilities, renal tubular acidosis	
*CASR*	Calcium-sensing receptor	AD 601199	Regulates PTH secretion and modulates calcium homeostasis	[25,26]
*CLCN5*	ClCN-5	X-linked 300009	Cl^-^/H^+^ exchanger	[27]
*CLCKNB*	CLCKNB	AR 607364	Chloride channel subunit, Bartter syndrome	[28]
*CLDN16*	Claudin-16	AR 248250	Claudin, paracellular Ca^2+^ and Mg^2+^ permeability	[29]
*CLDN19*	Claudin-19	AR 248190	Partners with cldn16; Claudin, paracellular Ca^2+^ and Mg^2+^ permeability	[30]
*CYP24A1*	Vitamin D 24-hydroxylase	AD/AR 143880	Hypercalcemia, degrades 1,25-OH_2_ Vit D_3_	[31]
*FAM20A*	Pseudokinase FAM20A	AR 204690	Amelogenesis imperfecta,	[32]
*FOXI1*	Foxhead Transcription factor I1	AR 601093	Regulates differentiation of acid-secretory intercalated cells	[33]
*GRHPR*	Glyoxylate reductase	AR 260000	Reduces glyoxylate; primary hyperoxaluria type 2	[34]
*HNF4A*	Hepatocyte nuclear factor 4α	AF 616026	Maturity-onset diabetes of the young, transcription factor regulating proximal and distal renal transporters	[35]
*HOGA1*	4-Hydroxy-2-oxoglutarate aldolase	AR 613616	Breaks down hydroxyproline; primary hyperoxaluria type 3	[36]
*HPRT*	Hypoxanthine phosphoribosyltransferase 1	X-linked recessive 300323	Purine metabolism, hyperuricemia	[37]
*KCNJ1*	ROMK potassium channel	AR 241200	Bartter syndrome	[38]
*MAGED2*	Melanoma antigen, family D, 2	X-linked recessive 300470	Regulator of SLC21A1, Bartter syndrome	[39]
*MEN1*	Menin1	AD 613733	Tumor suppressor in endocrine organs	[40,41]
*MOCOS*	Molybdenum cofactor sulfurase	AR 603592	Regulates xanthine dehydrogenase	[42]
*OCRL*	Inositol polyphosphate 5-phosphatase	X-linked recessive 300555	Dents type 2, PIP(2)) 5-phosphatase	[43]
*SLC12A1*	NKCC2	AR 601678	Na^+^/K^+^/2Cl^-^ cotransporter, Bartter syndrome	[44]
*SLC22A12*	URAT1	AR 220150	Hypouricemia, Uric acid transporter	[45]
*SLC26A1*	SAT1	AR 167030	Oxalate/sulfate exchanger	[46]
*SLC2A9*	Glut9	AD/AR 612076	Hypouricemia, uric acid transporter	[47]
*SLC34A1*	NaPi-IIa cotransporter	AR (AD ?) 616963	Hypercalcemia, phosphate-transporter	[48]
*SLC34A3*	NaPi-IIc cotransporter	AD/AR 241530	Hypercalcemia, rickets, phosphate-transporter	[49,50]
*SLC3A1*	rBAT	AR 220100	Cystine transporter subunit	[51]
*SLC4A1*	AE1 anion exchanger	AR/AD 611590	bicarbonate-chloride exchanger; dRTA and erythrocyte abnormalities	[52]
*SLC7A9*	b^0,+^AT1	AD/AR 220100	Cystine transporter subunit	[53]
*SLC9A3R1*	NHERF1	AD 612287	Scaffold protein for phosphate and urate transporters	[54]
*VDR*	Vitamin D receptor	AD 277440	Rickets, mediates calcitriol signaling	[55]
*WDR72*	WDR72	AR 613211	Amelogenesis imperfecta, dRTA, scaffold protein	[56,57]
*XDH*	Xanthine dehydrogenase	AR 278300	Degrades xanthine	[58]

List of genes involved in monogenic forms of kidney stone disease. OMIM numbers refer to disease caused by genetic variants in genes. The mode of inheritance is described as AD autosomal dominant, AR autosomal recessive, or as X-linked (recessive). The function of genes is mentioned and the first identification of variants referenced.

## THE CHALLENGE OF GENETIC DIAGNOSIS OF KIDNEY STONE DISEASE

Diagnosing genetic forms of kidney stone disease can be challenging. The occurrence of KSD in newborns, infants, adolescents and young adults (less than 25 years of age) points to a likely genetic cause if no other major kidney stone risk factors are present. The relatively low rate of detection of genetic causes of kidney stone disease in adult cohorts (with detection rates usually below 10%) may be caused by several issues. First, no genetic cause may be detected in patients that harbor nongenetic risk factors such as bariatric surgery, bowel resection, drinking low fluid volumes, or other life-style factors. Second, depending on the method of genetic testing, existing genetic variants may be missed. This may happen when using genetic panels not covering all known genes or only some relatively common variants. Many laboratories now use whole-exome sequencing, which usually covers the entire coding region of the genome but may miss genetic variants that lie in intronic regions. Also, it may be more difficult to detect compound heterozygous carriers, which may require more detailed analysis to assign genetic variants to the two alleles of the gene. A third problem is to decide whether genetic variants found in patients are causative for KSD. Classification of genetic variants usually follows the system of the American College of Medical Genetics and Genomics (ACMG) that classifies genetic variants into five main categories based on a framework designed to guide the interpretation of sequence variants in the clinical setting. These categories reflect the strength of evidence regarding a variant's impact on disease. Pathogenic (P) variants that are strongly supported by evidence to cause disease, likely pathogenic (LP) that have significant evidence suggesting disease causation, but not enough to be certain, variants of uncertain significance (VUS) with insufficient or conflicting evidence regarding their role in disease, Likely Benign (LB) and Benign (B), variants that most likely or certainly do not cause disease based on available evidence [59]. In many instances, where variants are classified as VUS the clinical challenge is that their unclear connection to disease hampers decision-making and guidance for patients and families. VUS findings provide neither a definitive diagnosis nor reassurance of benignity, which can lead to uncertainty in management, unnecessary tests or procedures, psychological distress, and the allocation of healthcare resources to ongoing reinterpretation efforts [60,61].

The interpretation of VUS may be facilitated by linking it to kidney stone analysis and metabolic work-up of patients including parameters in 24-h urine and blood providing the expected phenotype consistent with the gene variant [14^▪▪^]. However, the clinical utility of this approach has to be validated.

The likelihood of obtaining a molecular genetic diagnosis for KSD patients may be increased by preselecting patients for genetic diagnosis. Patients with a positive family history, age of first onset below 25 years, bilateral stone disease, or highly recurrent stone without other reason may be more likely to yield a genetic diagnosis [20].

## MONOGENIC KIDNEY STONE DISEASE IS A RISK FACTOR FOR HIGH RECURRENCE RATE AND LOSS OF KIDNEY FUNCTION

The role of common forms of kidney stone disease in the risk to develop chronic kidney disease (CKD) varies between cohorts [62,63,64–66^▪▪^]. However, monogenic kidney stone disease is strongly associated with both a high risk of stone recurrence and a substantially increased risk of eGFR decline and chronic kidney disease, especially when diagnosis and targeted therapy are delayed.

Overall, stone recurrence risk is high in KSD, with 10-year symptomatic recurrence rates of roughly 12–56% in unselected cohorts; monogenic diseases tend to cluster at the upper end of this spectrum or exceed it. For example, cystinuria, the most common monogenic stone disorder, causes recurrent cystine or mixed stones from childhood or early adulthood, often with multiple procedures; untreated patients can form new stones annually, and recurrence remains frequent even with standard measures such as high fluid intake, alkalinization, and thiol drugs. Primary hyperoxaluria, particularly type 1, is characterized by recurrent calcium oxalate stones and/or nephrocalcinosis with marked hyperoxaluria; many patients experience repeated stone events and interventions before diagnosis, and stone activity may slow but rarely ceases without causal therapy [67–69]. Several tubular disorders also show high recurrence. In Dent disease (*CLCN5* or *OCRL*), hypercalciuric nephrolithiasis and nephrocalcinosis are typical, and many affected males undergo multiple stone-related procedures in childhood and early adult life. Familial hypomagnesemia with hypercalciuria and nephrocalcinosis (CLDN16/19) and distal renal tubular acidosis due to *ATP6V1B1/ATP6V0A4/SLC4A1* pathogenic variants similarly present with recurrent calcium-based stones and medullary calcifications, often years before overt CKD is recognized [70,71].

Compared with common (polygenic) stone disease, patients with monogenic kidney stone disease present earlier, have higher recurrence rates, and show a much higher lifetime incidence of CKD and kidney failure due to recurrent obstruction, nephrocalcinosis, surgery, and crystal-associated tubulointerstitial damage [69,72]. Patients with cystinuria (defects in *SLC7A9* or *SLC3A1*) have a higher risk for loss of kidney function than a comparable cohort of patients with calcium oxalate stones and the number of open surgical procedures for stone removal contribute to this risk [73].

Likewise, in patients with kidney stones due to genetic variants in *SLC34A3,* a more rapid decline of eGFR and higher risk of CKD have been observed in various cohorts [74,75^▪▪^]. Also, patients with stones due to variants in *CLCN5* and *ORCL* (Dent's disease), with primary hyperoxalurias (*AGXT, HOGA1, GRHPR*) [26], in *APRT* (2,8-dihydroxyadenine crystal nephropathy) [76] have an increased risk to develop CKD.

Early identification of patients with monogenic kidney stone disease may thus help to reduce risks for complications and consequences such as CKD [75^▪▪^], particularly in forms of monogenic kidney stone disease where specific treatments are available (see the following section).

## IMPORTANCE OF SCREENING FOR EXTRARENAL MANIFESTATIONS OF MONOGENIC KIDNEY STONE DISEASE

Another important aspect of genetic diagnosis in monogenic KSD is the expression of some genes outside the kidney, as well as clinical manifestations in organs other than the kidney. For example, some forms of distal renal tubular acidosis are associated with extrarenal manifestations, such as sensorineural deafness (*FOXI1* and *ATP6V1B1*, and to a lesser extent, *ATP6V0A4* and *SLC4A1*), or amelogenesis imperfecta (*WDR72*) [71]. Similarly, patients with *BSND* variants develop sensorineural deafness. Carbonic anhydrase II (CAII) variants also cause osteopetrosis and intracranial calcifications. Patients with primary hyperoxaluria may develop disease in several extrarenal organs, most notably the heart (arrhythmias), bones (anemia and fractures), and skin (ulcers) [77]. Hepatocyte nuclear factor 4A (*HNF4A*) is also associated with maturity-onset diabetes (MODY) and Fanconi syndrome; however, only one variant (R76W) has been associated with KSD. *OCRL* is associated with intellectual disabilities and ocular manifestations. Variants in the tumor-suppressor *MEN* can cause kidney stones and tumors in multiple endocrine organs. Regular screening of patients with *MEN* variants may help with early detection of tumors and endocrinopathies. Genes involved in major metabolic pathways, such as *HPRT* and *XDH*, can cause multiple extrarenal manifestations, including seizures, learning disabilities, and failure to thrive in children. Early recognition of gene variants can help guide therapies and supportive measures for patients. Also, screening of additional family members may allow early diagnosis and prevention of disease in carriers. Conversely, the occurrence of kidney stones alongside extrarenal manifestations in children and young adults should prompt consideration of genetic causes and testing.

## THERAPY OF MONOGENIC KIDNEY STONE DISEASE

A last aspect of genetic diagnosis lies in the therapeutic consequences. Diagnosis of monogenic forms of KSD may improve therapy of patients as specific therapies are available for some forms [16^▪^]. These therapies may not only reduce stone recurrence rates but protect kidney function and may also improve extrarenal manifestations as most evident in patients with primary hyperoxaluria. Moreover, in patients with variants in the tumor-suppressor gene *MEN1*, surveillance of tumor development may be life-saving.

Tailored therapies are available for various forms of monogenic KSD as listed in Table 2.

**Table 2:** Available specific therapies for monogenic kidney stone disease genes

Gene(s)	Pathomechanism	Therapies	Ref.
*SLC4A1, ATP6V1B1, ATP6V0A4, FOXI1, WDR72*	dRTA with urinary acidification defect and metabolic acidosis	K-citrate or K-bicarbonate	[29]
*SLC34A1, SLC34A3*	Renal phosphate loss, hypervitaminosis D, secondary hypercalciuria	Avoid Vitamin D supplements, phosphate supplements	[34,78^▪▪^]
*CYP24A1*	Hypervitaminosis D	Avoid Vitamin D supplements and tanning beds, inhibitors of CYP24A1 (ketoconazole/fluconcazole), activators of CYP3A4 (rifampicin)	[31]
*CaSR*	Autosomal dominant hypocalcemia	PTH analogues, calcilytics	[79]
*SLC3A1, SLC7A9*	Cystinuria	Thiol-binding drugs (tiopronin, D-penicillamine), urine dilution and alkalinization	[80]
*AGXT, GRHPR, HOGA1*	Hyperoxaluria	AGXT: lumasiran, nedosiran, pyridoxine All variants: urine dilution, K-citrate or Mg-citrate	[36]
*SLC22A12, SLC2A9, HPRT*	Hyperuricosuria	Alkalinization of urine, allopurinol, febuxostat	
*XDH, MOCOS, MOCS1, MOCS2, GPHN*	Xanthinuria	Low purine diet	
*APRT*	Increased dihydroxyadenine excretion	Allopurinol, febuxostat	[35]

List of genes involved in kidney stone disease for which specific therapies are available. For details on genes and therapies, see text.

## CONCLUSION AND OUTLOOK

Genetic diagnosis allows identification of genetic variants that may cause kidney stone disease in a subset of patients. Careful preselection of patients may increase the rate of detection. Among these patients, newborn, children and adolescents below 25 years with stone disease are likely to have a higher rate of monogenic kidney disease. Similarly, genetic testing may also be considered in adult patients without any other obvious risk factor for stone disease. Careful metabolic diagnosis may provide additional evidence for forms of monogenic kidney disease. The relevance of monoallelic variants in classic monogenic kidney stone genes is becoming apparent and may increase diagnostic yield. However, careful clinical assessment including biochemical parameters in blood and urine may be needed to assure relevance of genetic findings. Genetic testing may also help guiding clinicians to consider extrarenal manifestations of genetic variants and may help to select patients for which specific therapies exist such as in forms of primary hyperoxaluria.

## Acknowledgements

*Figures have been created with Biorender.*

### Financial support and sponsorship

*Work by the authors has been supported by the Swiss National Science Foundation (C.A.W.), J.A.S. is funded by LifeArc, Medical Research Council (MR/Y007808/1), Kidney Research UK (Paed_RP_001_20180925, RP_007_20210729), the Northern Counties Kidney Research Fund (20/01) and the Gates Foundation.*

### Conflicts of interest

*C.A.W. reports honoraria from Kyowa Kirin, and collaborations with Bayer AG and NovoNordisk. M.S. reports honoraria from Avanzanite, Desitin and Anylam. J.A.S. has no conflicts of interest.*

## REFERENCES AND RECOMMENDED READING

Papers of particular interest, published within the annual period of review, have been highlighted as:

▪ of special interest
▪▪ of outstanding interest
